# Time-Dependent Nerve Growth Factor Signaling Changes in the Rat Retina During Optic Nerve Crush-Induced Degeneration of Retinal Ganglion Cells

**DOI:** 10.3390/ijms18010098

**Published:** 2017-01-05

**Authors:** Louise A. Mesentier-Louro, Sara De Nicolò, Pamela Rosso, Luigi A. De Vitis, Valerio Castoldi, Letizia Leocani, Rosalia Mendez-Otero, Marcelo F. Santiago, Paola Tirassa, Paolo Rama, Alessandro Lambiase

**Affiliations:** 1 San Raffaele Scientific Institute, Division of Neuroscience, Eye Repair Unit, 20132 Milan, Italy; lmesentier@biof.ufrj.br (L.A.M.-L.); l.devitis@studenti.unisr.it (L.A.D.V.); rama.paolo@hsr.it (P.R.); 2 Instituto de Biofísica Carlos Chagas Filho, Universidade Federal do Rio de Janeiro, Rio de Janeiro 21941-902, Brazil; rmotero@biof.ufrj.br (R.M.-O.); marcelo.santiago@biof.ufrj.br (M.F.S.); 3 National Research Council (CNR) Institute of Cell Biology & Neurobiology (IBCN), 00143 Rome, Italy; saradenicolo@libero.it (S.D.N.); pam.rosso@gmail.com (P.R.); paola.tirassa@cnr.it (P.T.); 4 Department of Science, University Roma Tre, 00146 Rome, Italy; 5 San Raffaele Scientific Institute, Division of Neuroscience, Institute of Experimental Neurology, 20132 Milan, Italy; castoldi.valerio@hsr.it (V.C.); leocani.letizia@hsr.it (L.L.); 6 Department of Sense Organs—Section of Ophthalmology, University of Rome “Sapienza”, 00185 Rome, Italy

**Keywords:** optic-nerve crush, retinal ganglion cells, nerve growth factor, glaucoma

## Abstract

Nerve growth factor (NGF) is suggested to be neuroprotective after nerve injury; however, retinal ganglion cells (RGC) degenerate following optic-nerve crush (ONC), even in the presence of increased levels of endogenous NGF. To further investigate this apparently paradoxical condition, a time-course study was performed to evaluate the effects of unilateral ONC on NGF expression and signaling in the adult retina. Visually evoked potential and immunofluorescence staining were used to assess axonal damage and RGC loss. The levels of NGF, proNGF, p75^NTR^, TrkA and GFAP and the activation of several intracellular pathways were analyzed at 1, 3, 7 and 14 days after crush (dac) by ELISA/Western Blot and PathScan intracellular signaling array. The progressive RGC loss and nerve impairment featured an early and sustained activation of apoptotic pathways; and GFAP and p75^NTR^ enhancement. In contrast, ONC-induced reduction of TrkA, and increased proNGF were observed only at 7 and 14 dac. We propose that proNGF and p75^NTR^ contribute to exacerbate retinal degeneration by further stimulating apoptosis during the second week after injury, and thus hamper the neuroprotective effect of the endogenous NGF. These findings might aid in identifying effective treatment windows for NGF-based strategies to counteract retinal and/or optic-nerve degeneration.

## 1. Introduction

Retinal ganglion cells (RGCs) of adult mammals do not regenerate spontaneously after axonal damage; and, similar to other central neurons, injury renders them unable to reestablish functional connections, with deleterious consequences for communication even between unaffected neurons [1,2,3]. RGC degeneration following optic-nerve crush (ONC) has been extensively described in mice and rats [4,5,6] and used as a model to study regenerative processes in the central nervous system [7,8,9]. Because RGCs are dependent on the innervating target, ONC might also be considered an appropriate model to investigate the consequences of reduced retrograde support of growth factors, including the nerve growth factor (NGF).

Following optic-nerve injury, indeed, cell death pathways are triggered by retrogradely transported signals from the lesion site and by the lack of trophic support from the glia and central targets [10,11]. Several growth factors are involved in RGC degeneration following optic-nerve injury [12]; among these, NGF has proven to play a role in the survival and growth of retinal neurons during development as well as in adult life and aging [13,14,15,16,17,18,19].

Paradoxically, RGC degeneration progresses even in the presence of a local increase of NGF. In glaucoma models, for example, retinal NGF is increased as early as seven days after induction of ocular hypertension [20,21]. These findings suggest that the enhancement of endogenous NGF levels is not sufficient to counteract neurotoxic mechanisms and support the survival of RGC, and that other events, probably affecting NGF receptor expression, might contribute to neurodegeneration [22].

The effect of NGF activity on target cells is mediated by two receptor types: tyrosine kinase A (TrkA), which selectively binds NGF, and p75-neurotrophin receptor (p75^NTR^), which can match to all neurotrophin family members [12], including neurotrophin precursor forms [23]. In general, NGF binding to TrkA leads to neuronal survival [18,19], while the activation of p75^NTR^ signaling is involved in the regulation of cell death, both during early retinal development [13,14,15] and in adulthood [16,17]. In recent years, proNGF has been shown to exert biological activity, and to mediate and regulate cell death by activating apoptotic signals after binding to p75^NTR^ and activating the JNK pathway [24,25]. Therefore, increased levels of proNGF, together with unbalanced levels of TrkA and p75^NTR^, are currently considered part of a pathological cycle that induces neuronal degeneration [26,27,28].

Whether the progressive RGC loss following optic-nerve injury might be related to dynamic changes in the expression of NGF and its receptors in the retina is not fully known. To address this question, a time-course study was performed to investigate the expression of NGF, proNGF and their receptors, and the intracellular signaling pathways activated by ONC in the retina. Histological and electrophysiological analyses were also performed to monitor RGC survival, axonal damage and visual function during the experimental course.

## 2. Results

### 2.1. Time-Course Effect of Nerve Crush on NGF, proNGF and Receptor Expression in the Retina

**Figure 1:**
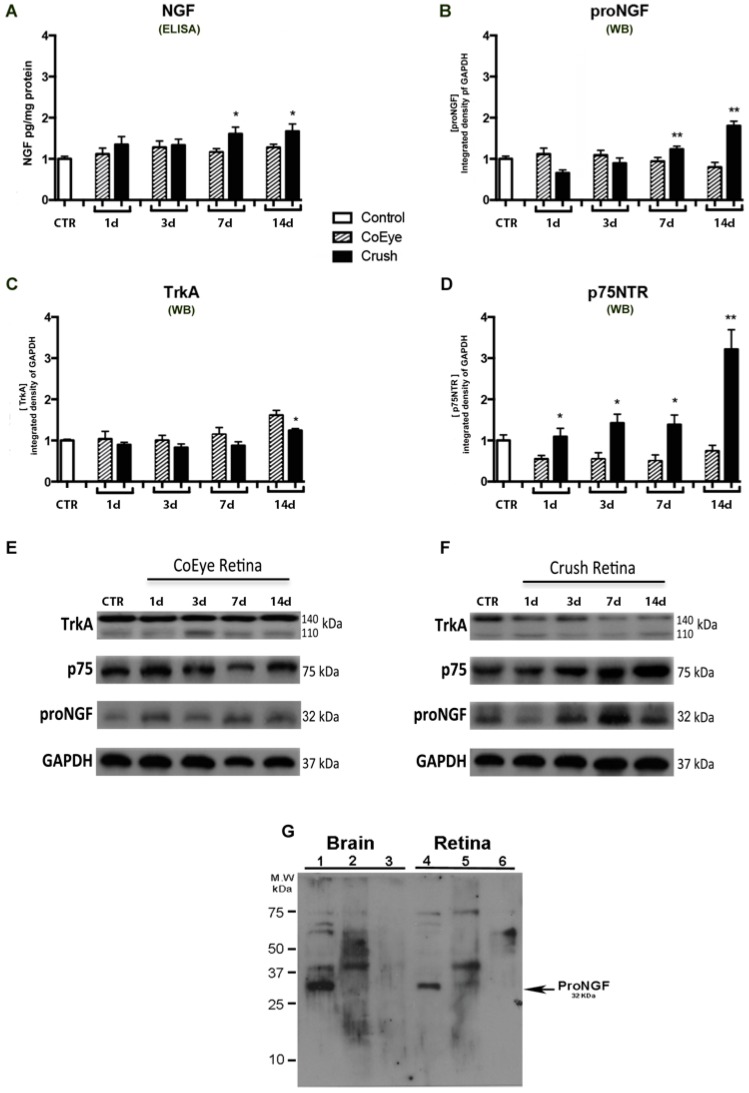
(**A**–**G**) Biochemical analysis of NGF, TrkA and p75^NTR^ in the retina from 1 to 14 days after crush (*n* = 6 per group). Crush retinas had increased levels of NGF anylate measured by Elisa (**A**) and proNGF quantified by WB analysis (**B**) at 7 d and 14 d. TrkA expression was reduced in Crush retinas at 14 d (**C**) while p75^NTR^ expression was increased in Crush retinas at all time points analyzed (**D**). Representative images of the WB of the time course in CoEye (**E**) and crushed retina (**F**). (**G**) The specificity of proNGF signal detected in both brain and retinal lysates using anti-proNGF by Alomone (Lanes 1 and 4). No specific signal was found in naïve samples (non reduced and denatured; Lanes 2 and 5), and by pre-incubation with the control peptide provided by Alomone (Lanes 3 and 6). * *p* < 0.05, ** *p* < 0.01. WB: Western blot; CoEye: contralateral eye; 1–14 d: 1 to 14 days after crush.

The expression levels of NGF (measured by ELISA), proNGF, p75^NTR^ and TrkA (evaluated by Western Blot (WB) analysis) in the retina of control (CTR, naïve), CoEye (eyes that were contralateral to crushed nerves) and Crush groups (nerve-crushed eyes) are reported in Figure 1. No significant changes were found by comparing the levels of each marker in the retina between the CTR (Figure 1, white bars) and CoEye (Figure 1, bars with oblique lines) groups at 1, 3, 7 and 14 days after crush (dac). In contrast, significant time-dependent variations were found in the Crush group (Figure 1, black bars) compared to CTR or CoEye.

A significant increase of NGF analyte measured by Elisa was found in the Crush retinas at 7 and 14 dac when compared to both CTR and CoEye, while no significant changes were detectable at 1 and 3 dac (Figure 1A). Similar results were obtained by WB analysis using the proNGF antibody. (see Figure 1B). No signal for the mature NGF form (about 13 kDa) was detectable in our WB conditions.

Analysis of the effect of nerve crush on the expression of NGF receptors revealed a different trend. No significant changes of TrkA expression were found in the first week after crush, but a significant decrease in the Crush retinas was observed at the end of the second week (*p* < 0.05; Figure 1C). Inversely, p75^NTR^ levels started to increase significantly from the first dac and reached the highest levels at 14 dac (Figure 1D).

### 2.2. Intracellular Pathway Activation in the Retina Following Nerve Crush

The activation of 18 signaling molecules in the retina was analyzed by a slide-based antibody array, which allows the detection of cellular proteins only when phosphorylated or cleaved at the specified residues. ERK1/2 (Thr202/Tyr2049) and BAD (Ser112) were phosphorylated in all samples, with no significant changes among the different groups. STAT3, S6 Rib Protein, HSP27, p70 S6 Kinase, p53 and GSK3 were not detectable in any of the samples, while STAT1, p38, SAPK/JNK and caspase-3 were activated only in Crush samples. The rest of the analyzed molecules were activated in both Crush and CoEye, showing different levels of activation depending on the time point, as reported in Table 1.

**Figure 2:**
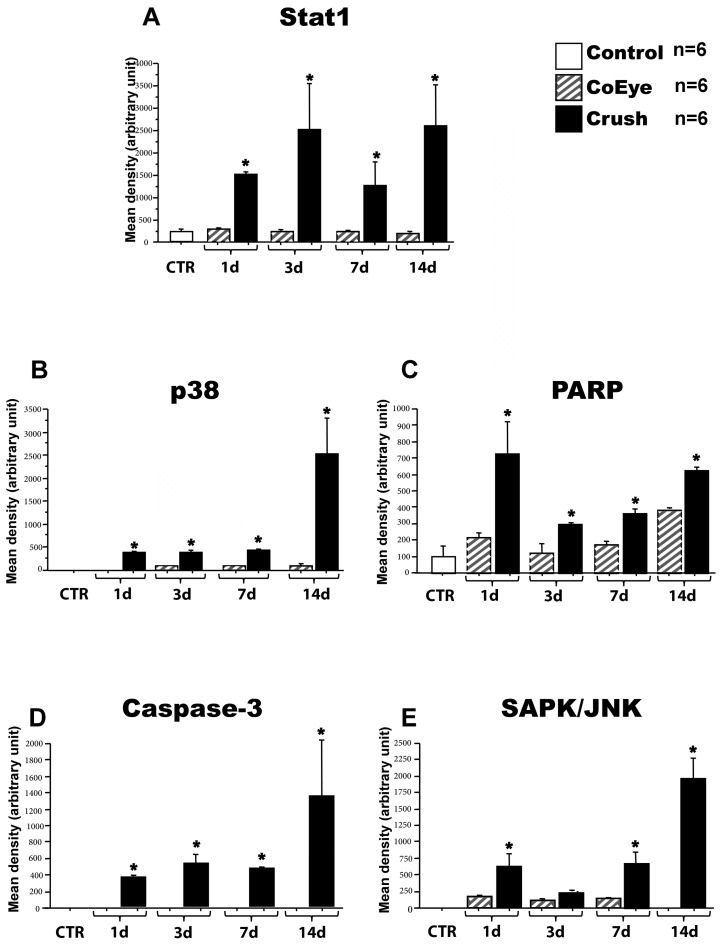
Intracellular pathways modulated in the retina 1 to 14 days after crush (*n* = 6 per group): (**A**) STAT1; (**B**) p38; (**C**) PARP; (**D**) Caspase-3; and (**E**) SAPK/JNK. * *p* < 0.05 vs. CoEye. CoEye: contralateral eye; 1–14 d: 1 to 14 days after crush.

The activation was quantified as described in the Materials and Methods Section. The statistical analysis showed that optic-nerve crush significantly increased the retinal levels of STAT1, p38, SAPK/JNK, caspase-3 and PARP (Figure 2).

### 2.3. GFAP and p75^NTR^ Expression in the Retina

**Figure 3:**
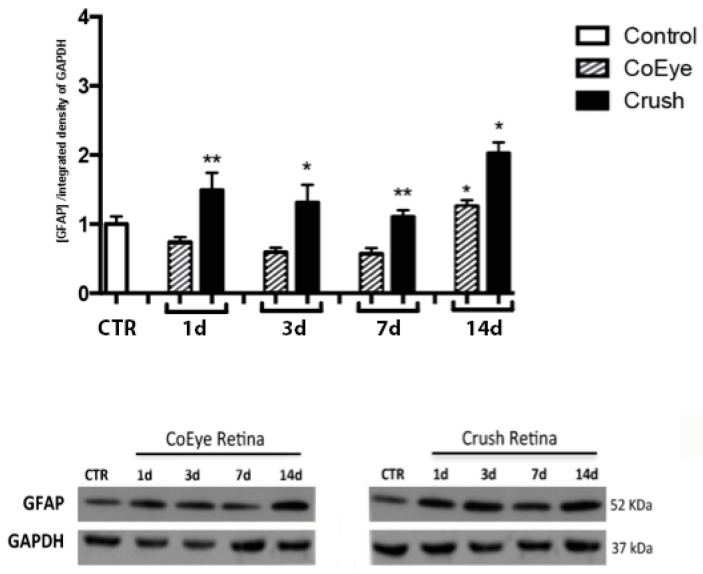
GFAP expression in the retina 1 to 14 days after crush. GFAP expression was increased in the Crush retinas at all time points analyzed (*n* = 6 per group). Lower panels show representative images of the WB. * *p* < 0.05, ** *p* < 0.01. CoEye: contralateral eye; 1–14 d: 1 to 14 days after crush.

GFAP levels were significantly increased in the retina in response to ONC (Figure 3, black bars), and the post-hoc analysis confirmed the effect of nerve crush when compared to the CTR (white bar) and CoEye (bars with oblique lines) at the different time points. No changes in GFAP expression levels were observed in the CoEye retina when compared to CTR.

**Figure 4:**
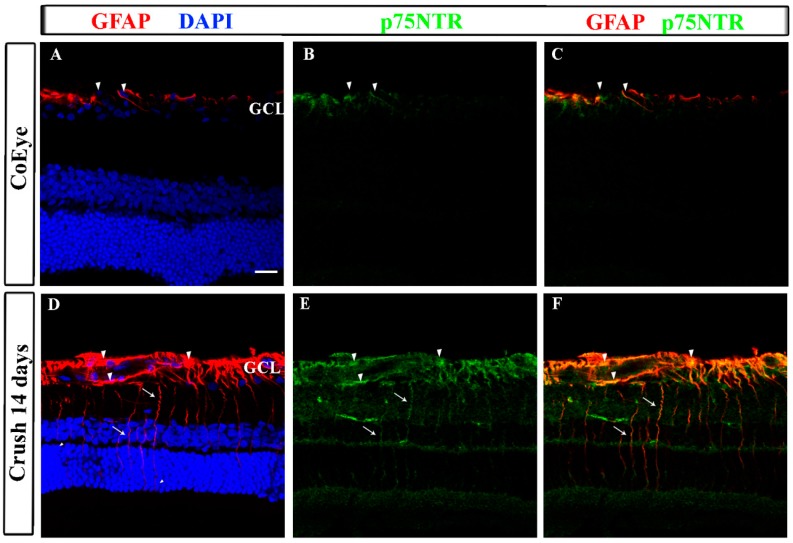
GFAP and p75^NTR^ expression in the retina 14 days after crush. Retinal sections of CoEye and Crush eyes. In the CoEye retinas, GFAP and p75^NTR^ are expressed only in the cells of the GCL (**A**,**B**). In the Crush retinas, astrocytes in the GCL and Müller cells (arrows indicate radial processes) up-regulate GFAP (**D**) and p75^NTR^ (**E**). (**C**,**F**) GFAP and p75^NTR^ merges. Arrowheads indicate cells that co-express GFAP and p75^NTR^. GCL: ganglion cell layer; scale bar: 20 μm. CoEye: contralateral eyes.

The histological analysis confirmed the biochemical data showing an increased expression of GFAP at 14 dac. In the CoEye, GFAP^+^ and p75^NTR+^ cells were mostly confined to the ganglion cell layer (GCL, Figure 4A,B), while in the Crush retinas both proteins were strongly expressed in the GCL and inner retinal layers. GFAP^+^ cell processes were distributed throughout the inner plexiform and nuclear layers (Figure 4B, arrows), indicating the presence of reactive astrocytes and increased GFAP expression in Müller cells. In addition, GFAP and p75^NTR^ expression were often co-expressed in the cells of the GCL and in Müller processes across the plexiform layers (Figure 4, arrowheads), suggesting that glial cells express this receptor in the retina.

### 2.4. Effects of ONC on the Functional and Structural Integrity of the Retina

**Figure 5:**
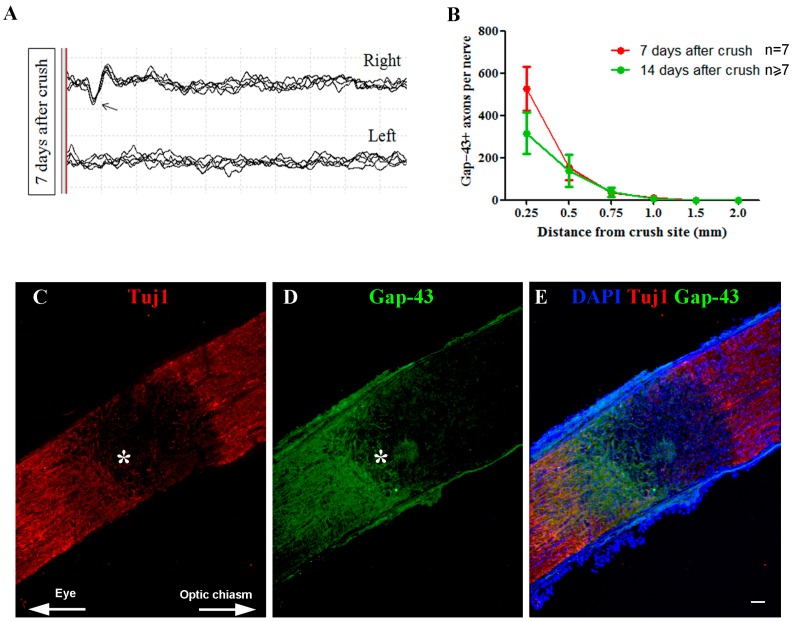
Functional and morphological aspects of the optic nerve after crush injury. (**A**) VEP recording of the CoEye (**right** eye) and Crush eyes (**left** eye), seven days after the unilateral crush procedure. **Upper** panel shows the right eye (CoEye) graph, where the arrow indicates the potential P1; **Lower** panel shows the left eye (Crush) graph, where no potential P1 was evoked; (**B**) Quantification of the number of Gap-43^+^ axons extending from 0.25 to 1.00 mm beyond the injury site, at 7 and 14 dac; (**C**) Optic nerve expression of Tuj1 at 7 dac; the asterisk indicates the proximal border of the injury site; (**D**) Gap-43+ expression is arrested at the injury site; (**E**) Merge with DAPI (blue). Scale bar: 50 μm. VEP: visually evoked potentials; CoEye: contralateral eye; dac: days after crush.

The in vivo recording of optic-nerve activity at 7 dac showed that regular visual potentials (Figure 5A, arrows indicate P1) were evoked after light stimulation of the CoEye, while the Crush eye did not respond to the light stimuli, indicating unilateral blindness caused by the nerve damage.

In addition, in the Crush eye, Tuj1 expression was interrupted approximately 1 mm from the optic disc, indicating the injury site (Figure 5C, asterisk), where Gap-43^+^ regenerating axons were mostly arrested (Figure 5D, asterisk). Similar functional impairment and histological damage were observable at 14 dac (data not shown).

Quantitative analysis confirmed the histological observations, showing a drastic reduction of Gap-43^+^ axons beyond the injury site (Figure 5B). Chiefly, at 7 dac, the number of axons decreased progressively. We found 526.6 ± 105.3 axons per nerve at 0.25 mm beyond the injury site, 153.6 ± 60.51 at 0.50 mm, 35.28 ± 11.74 at 0.75 mm, and 11.64 ± 5.029 at 1.00 mm. The number of regenerating axons was similar at 14 dac, when 316.4 ± 99.25 axons per nerve were found at 0.25 mm beyond the injury site, 139.9 ± 75.47 at 0.50 mm, 37.95 ± 22.71 at 0.75 mm, and 7.556 ± 5.049 at 1.00 mm. No Gap-43^+^ axons were found at 1.5 and 2.0 mm from the crush site at both 7 and 14 dac.

**Figure 6:**
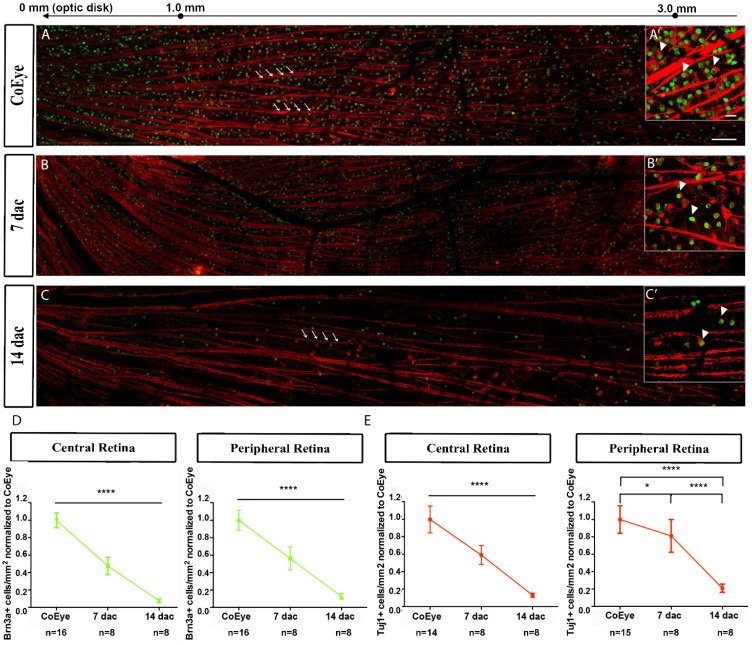
Expression of Tuj1 (red) and Brn3a (green) in the retina at 7 and 14 days after crush: (**A**) CoEye; (**B**) 7 dac; and (**C**) 14 dac. Arrows and arrowheads indicate axonal bundles and cell bodies, respectively; (**D**) Quantification of Brn3a^+^ cells in the central and peripheral retina; (**E**) Quantification of Tuj1^+^ cells in the central and peripheral retina. Scale bar: (**A**–**C**) 100 μm; (**A’**–**C’**) 20 μm. CoEye: contralateral eye; dac: days after crush. * *p* < 0.05; **** *p* < 0.0001.

The effects of nerve crush on the distribution of RGC are shown in photomontages of retinal images (Figure 6A–C), which show several Brn3a^+^ (green) nuclei and Tuj1^+^ (red) cell bodies and axon bundles. While in the CoEye (Figure 6A) a large number of Brn3a^+^ and Tuj1^+^ cells (A′, arrowheads) and axonal bundles (A, arrows) are visible, a progressive reduction of stained cells and axons is observable at 7 (Figure 6B) and 14 dac (Figure 6C,C′, arrows and arrowheads).

The time-dependent effect of ONC on RGC survival was further demonstrated by quantitative analysis. At 7 dac, the number of Brn3a^+^ cells in the central and peripheral retina was 50% of those detectable in the CoEye (Figure 6D), and the Tuj1^+^ cells were reduced on about 40% and 20% in the central and peripheral retina, respectively (Figure 6E).

In accordance with the axon damage, a more dramatic reduction of RGCs was found at 14 dac, when the Brn3a^+^ and Tuj1^+^ cells were reduced on more than 80% (Figure 6D–E). Raw data are reported in Table S1.

## 3. Discussion

The present study confirms and extends the information on the involvement of NGF in the optic nerve-injury model, showing that the progressive structural and functional alterations induced by nerve crush within two weeks are associated with time-dependent changes in NGF signaling. We found that retinal levels of the p75NTR receptor were enhanced immediately after injury and remained high at all time points examined, while no changes of TrkA expression levels were detected in the first week after crush, while a small but significant decrease was observed at 14 dac.

An increase of both NGF levels detected by Elisa, and proNGF analyzed by Western blot was also found at 7 and 14 dac. Recently, Malerba and colleagues [29] reported that some commercial NGF Elisa Kits might not efficiently discriminate between the mature and pro NGF and that the relative amount of the two NGF forms present in a sample might interfere with measurement outcome. Although the antibodies included in the Elisa Kit we used have a high affinity for rat β-NGF, it is possible that the high levels of proNGF in ONC retina samples (measured using WB experimental conditions set up to increase the detection of 32 kDa proNGF band) might have affected the selectivity and/or accuracy of NGF Elisa cannot be excluded. The NGF antibody produced by Alomone can detect a band corresponding at 13 kDa in retina [30], but due to our selective WB condition, which does not allow us to measure this NGF form in our samples, the time course effect of ONC on the mature NGF levels remain to be determined. Thus, only the effect of ONC on proNGF is speculated herein.

In addition, several intracellular pathways related to cell stress and death, such as PARP, Caspase-3, p38, STAT1 and SAPK/JNK, were up-regulated in the retina as soon as one day after nerve crush, sustained and eventually increased until 14 dac. In line with previous studies showing that the modulation of several pathways, including the activation of the apoptotic cascade, is observable in the retina as early as 12 h after optic-nerve injury [31,32], our data demonstrated that the rapid apoptotic induction following ONC, is associated with the increase of p75NTR expression, and precedes the enhancement of proNGF levels.

No changes in TrkA expression during the first week were found, whereas we observed a small but significant TrkA reduction in the second week after nerve crush, when more than 70% of RGCs are lost. Cui and coworkers found that the retinal levels of TrkA increase during the first five days post-axotomy but then decline during the subsequent three weeks, as RGC death progresses [33], supporting the concept that the expression of TrkA is directly dependent on the number of surviving cells. Actually, an injury-induced TrkA increase was also observed in the retina 48h after nerve axotomy [16]. However, this endogenous increase is not sufficient to protect RGC, while selective activation of TrkA affords RGC neuroprotection in a glaucoma model or following optic-nerve axotomy [22,34]. In contrast to unilateral ONC, a rapid neuronal degeneration is produced by axotomy and about 50% of the RGCs are lost in the first four days [4], suggesting that partial or complete nerve transection or impairment might differentially affect NGF receptor expression in RGCs.

As previously reported, impaired axonal transport and global axonal damage is produced as a consequence of the nerve compression in unilateral ONC, as early as 4 dac [35]. In this study, we found that although Gap-43-positive axons stopped at the injury site, Tuj1 was still expressed by distal axons at 7 dac, indicating a defective regeneration but an incomplete axonal degeneration at that time. This is consistent with the slow Wallerian degeneration that occurs in the central nervous system (CNS) [36].

Since apoptotic markers are up-regulated early in our experimental model, it is likely that the ONC-induced impaired connection with brain targets is sufficient to activate the cell-death program, although the RCG number declines more slowly than after complete axotomy or bilateral nerve crush. Indeed, only half the number of RGCs is lost within the first week after crush, while a further cell disappearance is observable in the following week.

Interestingly, while at 7 dac the TrkA levels are not altered, two weeks after ONC high proNGF and p75^NTR^ but low levels of TrkA were found in the retina, suggesting that the time point of TrkA expression reduction and/or imbalance of TrkA/p75^NTR^ is crucial for neuronal survival.

Lebrun-Julien and coworkers reported that RGC death following proNGF up-regulation is mediated by glial cells and that RGCs do not express p75^NTR^, which is, rather, expressed by Müller cells. The activation of p75^NTR^ in glial cells stimulates the production and release of TNF-α, leading to RGC death [17]. The idea that the up-regulation of p75^NTR^ in glial cells might contribute to RGC cell death after ONC is supported by our findings that the expression of p75^NTR^ and GFAP is increased in the retina starting from the first day post-crush and during the following 14 days, and that p75^NTR^ and GFAP are co-expressed in both cells and cell processes located in the GCL and in the inner nuclear layer. The damaging effects of increased p75^NTR^ levels, in both neurons and glia, have been extensively discussed in recent decades [37], although selective activation of this receptor by ligands that interact with specific domains induced the survival of peripheral and central neurons [38,39]. Lönngren and coworkers analyzed the mRNA levels of several growth factors and receptors in the rat retina following ischemia, finding that p75NTR mRNA is upregulated as soon as 12 h after injury [40], in agreement with the increased protein levels observed as early as 1 dac in the present study. Nevertheless, it cannot be excluded that other cell types, including microglia and blood-borne macrophages, which are activated during traumatic and degenerative CNS disorders [41], might also be involved in the p75^NTR^-mediated neurodegeneration.

Recently, Balzamino and coworkers (2015) showed that the levels of both NGF and p75^NTR^, but not TrkA, are increased in RGCs and bipolar cells in reelin-deprived retinas, a condition characterized by impaired retinal structure and visual function [42]. The expression of p75^NTR^ has also been observed in post-mitotic RGCs in the developing retina [15], indicating a role for this receptor and its activated signaling in the retinal cell fate during development. Together, these studies indicate a role for the unbalanced expression of NGF receptors when retinal homeostasis is altered, and suggests a crucial role for p75^NTR^ in retinal cell loss during development and after injury. However, further investigations are necessary to explore the possibility that ONC might influence the expression of p75^NTR^ in other cells than Müller glia and astrocytes in the retina.

## 4. Materials and Methods

### 4.1. Animals and Study Design

Adult Long Evans rats (male, 300–350 g) purchased from Charles River (Charles River Laboratories Italia s.r.l., Calco, Italy) were used in this study, in accordance with the ARVO Statement for the Use of Animals in Ophthalmic and Vision Research, and after approval by the Animal Care and Use Committee of the San Raffaele Scientific Institute (IACUC 572, 3 May 2013). All procedures were performed under anesthesia with ketamine and xylazine, and every effort was made to minimize suffering.

One week after arrival from the supplier, the rats were broadly divided into two groups: the control naïve rats (CTR group), which were used to measure the basal levels of the proteins evaluated and the pathway activation; and the rats submitted to unilateral ONC (Crush group), following the procedure described below. The contralateral eyes (CoEye) of the Crush group rats were used as a second control. Rats of the Crush group were euthanized at different time points, i.e., 1, 3, 7 and 14 days after crush (dac) to evaluate the time-dependent changes occurring in the retina.

The effects of nerve crush on the structural and functional integrity of the retina were also evaluated by in vivo electrophysiology, and by morphological and biochemical techniques, as described in detail below.

### 4.2. Optic-Nerve Crush (ONC)

The unilateral ONC was performed as described previously [35,43,44]. The rats were anesthetized by intraperitoneal injection of ketamine (70 mg/kg) and xylazine (10 mg/kg). Oxybuprocaine 0.4% eye drops were used as a topical anesthetic, and ophthalmic eye ointment was applied to prevent dehydration and damage to the ocular surface. Under a stereoscopic microscope, the left optic nerve was accessed by an incision in the skin that covers the orbital bone. The nerve was exposed and the dural sheath surrounding it was cut longitudinally. Nerve crush was performed by compression with tweezers (Dumont #5, 45°, 0.05 mm × 0.01 mm tip; World Precision Instruments, Berlin, Germany) for 15 s, at 1 mm behind the sclera. The contralateral right nerves were untouched and served as a control. After the procedure, the incision in the skin was sutured and topical antibiotic eye drops (Levofloxacin 5 mg/mL) were applied to the cornea. Animals were placed in a recovery cage with a heat pad and given a subcutaneous injection of Carprofen (5 mg/kg) for postoperative analgesia. The integrity of the retinal blood vessels was evaluated by fundoscopic examination, and animals showing signs of compromised blood supply were excluded from the study. At the time of euthanasia, both the left and right eyes were enucleated; the retinas and nerves from the left eyes with a crushed nerve were termed Crush, while those from the right eyes with an intact nerve were termed CoEye.

### 4.3. In Vivo Recording of Visually Evoked Potentials after Optic-Nerve injury

Visual function was analyzed in vivo at 7 dac (*n* = 5) by recording visually evoked potentials (VEPs), as previously described [45]. Briefly, VEPs were recorded after 5 min dark adaptation, under intraperitoneal anesthesia with ketamine (40 mg/kg) and xylazine (5 mg/kg). For each VEP session, 5 trains of 20 flash stimuli of 10 μs duration and 1 Hz frequency were delivered with a flash photostimulator (intensity 126–231 mJ; Micromed, Mogliano Veneto, Italy) placed 15 cm from the eye, with a 10–80 Hz bandpass filter.

### 4.4. Retina Dissection and Protein Extraction for Biochemical Analysis

Six CTR rats and 24 Crush rats (6 per time point) were deeply anesthetized with an overdose of ketamine and xylazine and euthanized by cervical dislocation. The eyes were removed and the right (CoEye) and left retinas (Crush) were quickly dissected on ice and stored in clean sterile tubes at −80 °C until use. To extract proteins, the retinal samples were homogenized by ultrasonication in buffer pH 7.00 containing 20 mM Tris-acetate pH 7.5, 150 mM NaCl, 1 mM EDTA, 1 mM EGTA, 2.5 mM sodium-pyrophosphate, 1 mM orthovanadate, 1 mM (*R*)-glycerolphosphate, 100 mM NaF, 1 mM phenylmethylsulfonyl fluoride and 1 g/mL leupeptin; kept in a cold room on a rotating shaker for 1 h to allow complete tissue disaggregation and cell lysis; and then centrifuged at 10,000× *g* for 30 min at 4 °C. The supernatants were used for total protein concentration measured by the Biorad assay, ELISA and intracellular signal assays, and Western blot analysis as described below.

### 4.5. Western Blot Analysis

Sample protein extracts were used for Western blotting and analysis of tissue levels of GFAP (1:20,000 anti-rabbit; Abcam, Cambridge, UK), proNGF (1:500 anti-rabbit; Alomone, Jerusalem, Israel) and the NGF receptors TrkA (1:1000 anti-rabbit; Cell Signaling Technology, Danvers, MA, USA) and p75^NTR^ (1:1000 anti-rabbit; Cell Signaling Technology). Samples (20–50 μg total protein) were dissolved in loading buffer (0.1 mol/L Tris–HCl buffer, pH 6.8, containing 0.2 mol/L DTT, 4% SDS, 20% glycerol, and 0.1% bromophenol blue), separated by SDS-PAGE, and electrophoretically transferred to polyvinylidene fluoride (PVDF) or nitrocellulose membranes. The membranes were incubated for 1 h at room temperature with 5% non-fat dried milk dissolved in TBST (10 mmol/L Tris, pH 7.5, 100 mmol/L NaCl and 0.1% Tween-20), washed three times for 10 min each in TBST, and then incubated overnight at 4 °C with primary antibody. Horseradish peroxidase-conjugated anti-rabbit IgG (Cell Signaling Technology, Danvers, MA, USA) was used as a secondary antibody. The blots were developed with ECL Chemiluminescent HRP Substrate (Sigma, Darmstadt, Germany) as the chromophore. The public-domain ImageJ software (http://rsb.info.nih.gov/ij/) was used for gel densitometry. The band quantification was performed following the tutorial (http://lukemiller.org/index.php/2010/11/analyzing-gels-and-western-blots-with-image-j/) created to standardize the gel band quantification, to eleminate sample to sample/gel to gel variability. The integrated density of glyceraldehyde 3-phosphate dehydrogenase (anti-rabbit GAPDH-HRP conjugate antibody; Cell Signaling Technology) served as the normalizing factor. Six retinas were used for each group (CTR, Crush or CoEye), at each time point. Data are expressed as relative optical density (arbitrary units) and presented as means ± SEM.

### 4.6. Neurotrophin Concentrations

NGF concentration in the retina was measured by ELISA assay using the rat b-NGF Duoset Elisa (R&D Systems, Inc., Minneapolis, MN, USA), following the manufacturer’s instructions. The assay is specific for rat b-NGF with approximately 0.1% and 50% cross-reactivity with human and mouse recombinant b-NGF respectively, and has no cross-reactivity with other human or rat growth factors. The colorimetric reaction product was measured at 450 nm using a microplate reader (Dynatech MR 5000; PBI International, Dynatech International, Edgewood, NY, USA). Results are expressed as pg/mg protein (mean ± SEM).

### 4.7. Analysis of Activated Intracellular Signal Pathways

The PathScan Intracellular Signaling array kit (Cell Signalling Technology) was used, according to the manufacturer’s instructions, to detect the nerve crush-induced phosphorylation or cleavage of signaling molecules in the retina at different post-operation times. The kit allows the simultaneous detection of 18 signaling molecules when phosphorylated, including ERK1/2 (Thr102/Tyr204), Stat1 (Tyr701), Stat3 (Tyr705), AKT (Thr308), AKT (Ser473), AMPKa (Thr172), S6 Ribosomal Protein (Ser235/236), mTOR (Ser2448), HSP27 (Ser78), BAD (Ser112), p70S6K (Thr389), PRAS40 (Thr246), p53 (Ser15), p38 (Thr180/Tyr182), SAPK/JNK (Thr183/Tyr185) and GSK-3beta (Ser9); or cleaved, including PARP (Asp214) and Caspase-3 (Asp175), which allows one to investigate the activation of proteins involved in cell cycling, growth, and survival.

Protein samples were diluted in array diluent buffer to 1 mg/mL. Glass slides with antibody spotted nitrocellulose pads were connected with a multi-well gasket for blocking each pad with 100 µL array blocking buffer per well for 15 min, followed by 16 h incubation at 4 °C with 75 µL of diluted samples/well. After four washing steps with 100 µL of array wash buffer, the pads were incubated with 75 µL of the detection antibody cocktail for 1 h at RT. LumiGLO Reagent was used to reveal the bound detection antibody by chemiluminescence.

The images were captured with the UVItec gel documentation system (UVItec Limited, Cambridge, UK) and the spot intensities were quantified using the software Nikon NIS-Elements AR 2.30 (Nikon Instruments Europe BV, Amsterdam, The Netherlands). A fixed threshold over the background, and feature restriction functions were applied to define the measurable spot area and intensity. Measurements were standardized between the experimental groups using the same calibration system and threshold. Data are expressed as mean optical density (arbitrary units) and presented as mean ± SEM.

### 4.8. Histological Preparation and Immunohistochemistry

For histological evaluation of the optic nerve and retina, rats on the 7th (*n* = 8) or 14th (*n* = 8) dac were deeply anesthetized with an overdose of ketamine and xylazine and perfused through the heart with ice-cold saline, followed by 4% paraformaldehyde (pH 7.4). The eyes and optic nerves were cleaned of connective tissue and post-fixed in 4% paraformaldehyde for 2 h at 8–10 °C

The retinas were dissected and processed for whole-mount preparations. Retina whole-mount samples were processed for immunostaining detection of RGCs using specific antibodies to β-III tubulin (Tuj1) and Brn3a [35,46,47,48,49,50,51,52]. Primary antibodies anti-Brn3a produced in goat (1:250, Santa Cruz Biotechnology, Santa Cruz, CA, USA) and anti-Tuj1 produced in mouse (1:250, Covance Laboratories, San Diego, CA, USA) were diluted in PBS with 0.2% Triton X-100 (PBST) and 5% normal donkey serum (Sigma-Aldrich, St. Louis, MO, USA) and incubated overnight at 4 °C. Retinas were washed three times in PBS and incubated with the secondary antibodies Alexa 488 donkey anti-goat IgG and Alexa Fluor 546 donkey anti-mouse IgG (1:1000, Life Technologies, Camarillo, CA, USA) in the same solution as the primaries for 2 h at room temperature. The retinas were washed three times in PBS, flat-mounted, and covered with VECTASHIELD mounting medium (Vector Laboratories, Inc., Burlingame, CA, USA). All steps were performed under gentle shaking.

The eyes and optic-nerve segments used for GFAP, p75^NTR^, Tuj1 and Gap-43 were transferred to PBS with increasing sucrose concentrations until 30%. The tissue was embedded in optimal cutting temperature compound (Bio-Optica, Milan, Italy) and cut longitudinally on a cryostat (Leica Microsystems, Nussloch GmbH, Nussloch, Germany) at 14–20 µm thickness. Tissue sections were rinsed with 0.1% PBST and incubated with 5% normal donkey serum in the same solution for 30 min at room temperature to block non-specific binding. Primary antibodies anti-Gap-43 produced in rabbit (1:50, Santa Cruz Biotechnology, Santa Cruz, CA, USA), anti-β-III tubulin produced in mouse (Tuj1, 1:250, Covance Laboratories), anti-GFAP produced in rabbit (1:400, Abcam, Cambridge, UK) and/or anti-p75^NTR^ produced in mouse (1:100, Santa Cruz Biotechnology) were diluted in 0.1% PBST. Tissue sections were incubated with the primary antibodies overnight at 4 °C, washed in PBS, and then incubated with secondary antibodies Alexa Fluor 488 donkey anti-rabbit IgG and/or Alexa Fluor 546 donkey anti-mouse IgG (1:1000, Life Technologies) for 2 h at room temperature. Sections were then washed three times in PBS and mounted with VECTASHIELD with DAPI (Vector Laboratories).

Retina and nerve sections were analyzed by a blinded observer with the aid of a fluorescence microscope (Leica CTR5500; Leica Microsystems) and used for quantification as described below. Representative images were acquired with a confocal microscope (TCS SP5; Leica Microsystems).

### 4.9. Quantification of RGC Number

A total of 32 flat-mounted retinas (*n* = 16 for CoEye; *n* = 8 for Crush 7 dac; *n* = 8 for Crush 14 dac) were imaged by a blinded observer with the aid of a fluorescence microscope (Leica CTR5500; Leica Microsystems), with the focus positioned on the ganglion cell layer (GCL), as described previously [46,47]. Images were randomly acquired at approximately 1.0 mm and (central retina) and 3.5 mm (peripheral retina) from the optic disc, in all quadrants of the retina. Twenty images of 0.064 mm^2^ (40× magnification) were taken for Tuj1 staining, and 10 images of 0.366 mm^2^ (20× magnification) were taken for Brn3a, which because of its nuclear expression allows semi-automatic cell counting on images acquired in a lower magnification. A blinded observer manually counted Tuj1+ cell bodies. Brn3a images were converted to binary images and immunostained cells were automatically counted using the Analyze Particles function (Size 150-2000; Circularity 0.25–1.00) of ImageJ software (NIH). The mean number of cells was divided by the area and normalized by the CoEye. Data is expressed as mean ± SEM. Representative images of the retinas were acquired with a confocal microscope (TCS SP5; Leica Microsystems).

### 4.10. Quantification of Axon Outgrowth

The optic nerves were immunostained for Gap-43 and analyzed under a fluorescence microscope (Leica CTR5500; Leica Microsystems). The center of the field of a 40× magnification objective lens was positioned at 0.25, 0.50, 0.75, 1.00, 1.50 and 2.00 mm from the proximal border of the crush site. At each point, a blinded observer counted the number of Gap-43^+^ axons and measured the cross-sectional width of the nerve, in 5 longitudinal sections. The values obtained were normalized by the formula described by Leon and colleagues [53] and expressed as the total number of axons per nerve at each distance from the lesion site (mean ± SEM).

### 4.11. Statistical Analysis

Biochemical data were subjected to one-way ANOVA, using the SuperANOVA package for Macintosh (Abacus Concepts, Berkeley, CA, USA) considering the CTR, CoEye and Crush groups at the indicated time points as variables. Multicomparison analysis was performed by Tukey–Kramer post-hoc test.

The Tuj1 and Brn3a cell counts were compared among CoEye, at 7 and 14 dac, using a One-Way ANOVA with Tukey’s multiple comparisons test. Gap-43 counts were compared between CoEye and Crush groups using a Student t-test at each distance analyzed, using Prism 6.0 software (GraphPad, CA, USA).

## 5. Conclusions

Our study indicated that the imbalance of NGF receptors might be crucial during the post-injury phase, in which RGCs are dramatically lost, by increasing their vulnerability to endogenous mechanisms of cell death. Indeed, the finding that RCG death occurs in the presence of high levels of p75^NTR^ and low levels of TrkA led us to postulate that endogenous NGF does not encounter enough TrkA receptors to activate survival pathways and suggests a proNGF/p75^NTR^-mediated mechanism for ONC-induced RGC degeneration. This temporal course of changes in NGF signaling, associated with the level of retinal and optic-nerve degeneration, may be useful to develop therapeutic strategies to counteract the neuronal loss that occurs in response to axonal damage.

## Figures and Tables

**Table 1:** Time course effect of nerve crush on the activation of intracellular signaling molecules in the retina.

Intracellular Signals	CTR		1 dac		3 dac		7 dac		14 dac	
			CoEye	Crush	CoEye	Crush	CoEye	Crush	CoEye	Crush
ERK1/2	x	x	x	x	x	x	x	x	x	x
STAT1 *	–	–	–	x	–	x	–	x	–	x
STAT3	–	–	–	–	–	–	–	–	–	–
AKT (Thr308)	–	–	–	–	x	x ^a^	x	x ^a^	x	x
AKT (Ser473)	–	–	–	x ^a^	x	x	x	x ^a^	x	x
AMPKa	–	–	x	x	x	x	x	x	x	x
S6 Rib.Prot	–	–	–	–	–	–	–	–	–	–
mTor	–	–	x	x ^a^	x	x	x	x ^a^	x	x
HSP27	–	–	–	–	–	–	–	–	–	–
Bad (Ser 112)	x	x	x	x	x	x	x	x	x	x
p70 S6 Kinase	–	–	–	–	–	–	–	–	–	–
Pras 40	–	–	x	x ^a^	x	x	x	x	x	x ^a^
p53	–	–	–	–	–	–	–		–	–
p38 *	–	–	–	x	–	x	–	x	–	x
Sap/NJK *	–	–	–	x	–	x	–	x	–	x
PARP *	–	–	–	x	x	x	x	x	x	x
Caspase 3 *	–	–	–	x	–	x	–	x	–	x
GSK3	–	–	–	–	–	–	–	–	–	–

Only signal molecules with optical density >0.250 over the background level are reported in the table. The letter “x” indicates activation; the molecules indicated with an asterisk (*) in the “Intracellular signals” column were significantly activated by nerve crush at all time points considered, and their trend is shown in Figure 2. The letter “a” indicates *p* < 0.05 compared to the contralateral eye (CoEye). CTR: control, naïve retina; dac: days after crush.

## Supplementary Materials

Supplementary materials can be found at www.mdpi.com/1422-0067/18/1/98/s1.
