# Correction: Mesentier-Louro et al. Time-Dependent Nerve Growth Factor Signaling Changes in the Rat Retina During Optic Nerve Crush-Induced Degeneration of Retinal Ganglion Cells. *Int. J. Mol. Sci.* 2017, *18*, 98

**DOI:** 10.3390/ijms27104175

**Published:** 2026-05-08

**Authors:** Louise A. Mesentier-Louro, Sara De Nicolò, Pamela Rosso, Luigi A. De Vitis, Valerio Castoldi, Letizia Leocani, Rosalia Mendez-Otero, Marcelo F. Santiago, Paola Tirassa, Paolo Rama, Alessandro Lambiase

**Affiliations:** 1 San Raffaele Scientific Institute, Division of Neuroscience, Eye Repair Unit, 20132 Milan, Italy; lmesentier@biof.ufrj.br (L.A.M.-L.); l.devitis@studenti.unisr.it (L.A.D.V.); rama.paolo@hsr.it (P.R.); 2 Instituto de Biofísica Carlos Chagas Filho, Universidade Federal do Rio de Janeiro, Rio de Janeiro 21941-902, Brazil; rmotero@biof.ufrj.br (R.M.-O.); marcelo.santiago@biof.ufrj.br (M.F.S.); 3 National Research Council (CNR) Institute of Cell Biology & Neurobiology (IBCN), 00143 Rome, Italy; saradenicolo@libero.it (S.D.N.); pam.rosso@gmail.com (P.R.); paola.tirassa@cnr.it (P.T.); 4 Department of Science, University Roma Tre, 00146 Rome, Italy; 5 San Raffaele Scientific Institute, Division of Neuroscience, Institute of Experimental Neurology, 20132 Milan, Italy; castoldi.valerio@hsr.it (V.C.); leocani.letizia@hsr.it (L.L.); 6 Department of Sense Organs—Section of Ophthalmology, University of Rome “Sapienza”, 00185 Rome, Italy

In the original publication [1], there was a mistake in Figure 3 as published. The wrong representative picture of GAPDH expression in the CoEye was shown. The corrected Figure 3 appears below. The authors state that the scientific conclusions are unaffected. This correction was approved by the Academic Editor. The original publication has also been updated.

## Figures and Tables

**Figure 3:**
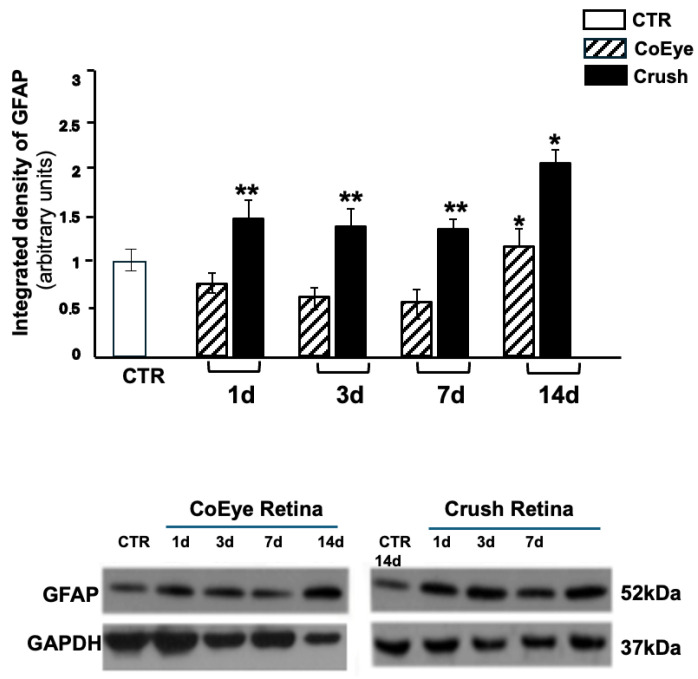
GFAP expression in the retina 1 to 14 days after crush. GFAP expression was increased in the Crush retinas at all time points analyzed (*n* = 6 per group). Lower panels show representative images of the WB. * *p* < 0.05, ** *p* < 0.01. CoEye: contralateral eye; 1–14 d: 1 to 14 days after crush.
