# Supplementary material for: Time-Dependent Nerve Growth Factor Signaling Changes in the Rat Retina During Optic Nerve Crush-Induced Degeneration of Retinal Ganglion Cells
Source: Int J Mol Sci. 2017 Jan 5;18(1):98. doi: 10.3390/ijms18010098 (PMC5297732; doi:10.3390/ijms18010098)
Supplement: Supplementary file 1 [file ijms-18-00098-s001.pdf]

Supplementary Materials: Time-Dependent Nerve Growth Factor Signaling Changes in the Rat Retina during Optic Nerve Crush-Induced Degeneration of Retinal Ganglion Cells

Louise A. Mesentier-Louro, Sara De Nicolò, Pamela Rosso, Luigi A. De Vitis, Valerio Castoldi, Letizia Leocani, Rosalia Mendez-Otero, Marcelo F. Santiago, Paola Tirassa, Paolo Rama and Alessandro Lambiase

Table S1. Number of Tuj1- and Brn3a-positive cells in the retina. Table shows the number of cells per square millimeter of retina and SEM. The number of experiments (*n*) is indicated at each point.

	**CoEye**			**7 dac**			**14 dac**		
**Central Retina**	**cells/mm²**	**SEM**	***n***	**cells/mm²**	**SEM**	***n***	**cells/mm²**	**SEM**	***n***
$Brn3a^+$	1842	39.24	16	876.7	64.86	8	138.3	12.79	8
$Tuj1^+$	1264	52.31	14	746.8	48.62	8	164.3	11.29	8
**Peripheral Retina**	**cells/mm²**	**SEM**	***n***	**cells/mm²**	**SEM**	***n***	**cells/mm²**	**SEM**	***n***
$Brn3a^+$	922.8	26.09	16	517.7	42.85	8	115.2	10.55	8
$Tuj1^+$	1170	47.75	15	949.8	77.81	8	243.7	19.68	8
